# Activated Hepatic Stellate Cells Induce Infiltration and Formation of CD163^+^ Macrophages *via* CCL2/CCR2 Pathway

**DOI:** 10.3389/fmed.2021.627927

**Published:** 2021-02-05

**Authors:** Sujuan Xi, Xiaoyan Zheng, Xiangyong Li, Yuming Jiang, Yuankai Wu, Jiao Gong, Yusheng Jie, Zhanyi Li, Jing Cao, Liuping Sha, Min Zhang, Yutian Chong

**Affiliations:** ^1^Department of Infectious Diseases, Third Affiliated Hospital of Sun Yat-sen University, Guangzhou, China; ^2^Key Laboratory of Tropical Disease Control, Ministry of Education, Sun Yat-sen University, Guangzhou, China; ^3^The Reproductive Medical Center, The Seventh Affiliated Hospital of Sun Yat-sen University, Shenzhen, China; ^4^Department of General Surgery, Nanfang Hospital, Southern Medical University, Guangzhou, China

**Keywords:** activated hepatic stellate cells, liver fibrosis, M2 macrophage, CCL2, hepatitis B

## Abstract

**Background:** Activated hepatic stellate cells (aHSCs) regulate the function of immune cells during liver fibrosis. As major innate cells in the liver, macrophages have inducible plasticity. Nevertheless, the mechanisms through which aHSCs regulate macrophages' phenotype and function during liver fibrosis and cirrhosis remain unclear. In this study, we examined the immunoregulatory function of aHSCs during liver fibrosis and explored their role in regulating macrophage phenotype and function.

**Methods:** A total of 96 patients with different stages of chronic hepatitis B-related liver fibrosis were recruited in the study. Metavir score system was used to evaluate the degree of fibrosis. The expression of hepatic CCL2 and M2 phenotype macrophage marker CD163 were detected by immunohistochemistry, and the relationship among hepatic CD163, CCL2, and fibrosis scores were also explored. In the *in vitro* model, the aHSCs isolated from human liver tissues and THP-1-derived M0-type macrophages (M0MΦ) were co-cultured to observe whether and how aHSCs regulate the phenotype and function of macrophages. To explore whether CCL2/CCR2 axis has a crucial role in macrophage phenotypic changes during liver fibrosis, we treated the M0MΦ with recombinant human CCL2 or its specific receptor antagonist INCB-3284. Furthermore, we used LX2 and TGF-β-activated LX2 to mimic the different activation statuses of aHSCs to further confirm our results.

**Results:** In patients, the infiltration of M2 macrophages increased during the progression of liver fibrosis. Intriguingly, as a key molecule for aHSC chemotactic macrophage aggregation, CCL2 markedly up-regulated the expression of CD163 and CD206 on the macrophages, which was further confirmed by adding the CCR2 antagonist (INCB 3284) into the cell culture system. In addition, the TGF-β stimulated LX2 further confirmed that aHSCs up-regulate the expression of CD163 and CD206 on macrophages. LX2 stimulated with TGF-β could produce more CCL2 and up-regulate other M2 phenotype macrophage-specific markers, including IL-10, ARG-1, and CCR2 besides CD163 and CD206 at the gene level, indicating that the different activation status of aHSCs might affect the final phenotype and function of macrophages.

**Conclusions:** The expression of the M2 macrophage marker increases during liver fibrosis progression and is associated with fibrosis severity. AHSCs can recruit macrophages through the CCL2/CCR2 pathway and induce M2 phenotypic transformation.

## Introduction

Liver cirrhosis is the eleventh most common cause of mortality worldwide, causing more than 1.16 million deaths annually (1, 2). The essence of fibrosis is the wound-healing response to chronic inflammation. When this process is dysregulated, excessive scarring occurs in response to persistent injury, leading to altered tissue functions (3, 4). The immune system has a dual role in liver fibrosis pathological process by mediating the immune-inflammatory reactions and causing liver damage (4). Impaired liver immune system surveillance, which is also the main pathological feature of cirrhosis, occurs primarily due to incomplete and inappropriate activation of immune cells or impaired response of the immune system to pathogens (5–7). Some studies have shown that immune cell, including monocytes/macrophages, NK cells, and lymphocytes, show impaired functions during cirrhosis (6, 8).

Macrophages featured for strong plasticity can differentiate into pro-fibrotic and anti-fibrotic macrophages responding to different conditions (9–11). These cells exert opposite functions by producing pro-inflammation or pro-fibrotic factors in different circumstances (11–14). Typically, the classically activated macrophages (M1) secrete the pro-inflammatory cytokines that exert anti-infective effects; alternatively activated macrophages (M2) mainly regulate the inflammatory response and tissue repair (12, 15). However, persistent inflammatory response in patients with chronic hepatitis suggests that liver immune cells' tolerance might impede effective immune surveillance in the liver (6, 7). Reportedly, peripheral blood monocytes/macrophages are mainly M2 phenotypes in the late stages of hepatitis B-related cirrhosis with the decreased anti-infectivity, thereby increasing the possibility of bacterial infection (16). In clinic, high M2-specific CD163 levels indicate poor prognosis; these levels are correlated with increased tumor nodules and venous infiltration in HCC patients (17). However, the mechanisms related to M2 macrophage formation during chronic liver diseases, especially liver fibrosis/cirrhosis, are not entirely clear.

In chronic liver injury, hepatic stellate cells resting in the sinus (the Disse) lumen receive signals from damaged hepatocytes, macrophages, and other immune cells and rapidly activate to myofibroblast-like cells, also known as activated hepatic stellate cells (aHSCs) (18–21). These cells display fibrogenic properties by producing excessive extracellular matrix and collagen (22). Recently, the unexpected immunoregulatory roles of aHSCs in liver fibrosis received increasing interest (8, 21). Our previous study demonstrated that aHSCs secrete many immunomodulatory cytokines and chemokines to regulate the phenotype and function of monocytes, NK cells, and T cells (23–25). It is important to continue exploring how aHSCs regulate macrophage's phenotype and function in the long process of liver fibrosis.

CCL2, also known as monocyte chemotactic factor 1 (MCP1), regulates the migration and infiltration of monocytes/macrophages through combination with its specific receptor CCR2 (26, 27). It has been reported that CCL2/CCR2 axis has a vital role during fibrosis (28–31). CCL2-dependent infiltrating macrophages promote angiogenesis in progressive liver fibrosis. CCR2 is mainly responsible for recruiting pro-inflammatory and profibrogenic infiltrating monocytes during fibrosis progression (29). Using liquid chip screening, we previously found that CCL2 may profoundly enrich in the supernatant of aHSCs (23). Yet, so far, only a few studies have explored whether CCL2 independently affects the phenotype and function of monocytes/macrophages in the process of liver fibrosis. In the present study, we found that aHSCs aggregate the macrophages through CCL2/CCR2 pathway and induce M2 phenotypic transformation during liver fibrosis.

## Materials and Methods

### Patients and Specimens

Liver tissues from different fibrosis stages were obtained from 96 patients who underwent curative liver resection of hepatocellular carcinoma at the Third Affiliated Hospital of Sun Yat-sen University in south China from 2014 to 2018. Patients with hepatitis C virus, HIV, Wilson's disease, autoimmune liver disease, genetic metabolic liver disease, and other undefined pathogenesis liver diseases were excluded from the study. The fibrotic tissues were excised at least 3 cm away from the tumor's edge, as previously described (15, 23, 32). Fresh fibrotic liver tissues from five patients were selected for the acquisition of primary aHSCs.

The experimental protocol was in accordance with the Helsinki Declaration and the local ethical guidelines. The study was approved by the Institutional Review Board of the Third Affiliated Hospital of Sun Yat-sen University.

### Immunohistochemistry and Immunofluorescence Staining

The different stages of liver fibrosis were evaluated according to the Metavir score system based on the hematoxylin-eosin staining. Paraffin-embedded and formalin-fixed samples were processed for IHC. The slices were probed with primary antibody targeted against human CD68, CD163 (Zsbio, China), and CCL2 (Sigma Aldrich, St. Louis, MO, USA), and stained with either diaminobenzidine or 3-amino-9-ethylcarbazole using the Envision System (Dako Cytomation, Glostrup, Denmark). Five representative fields were selected at 200× magnification using microscopy (Leica, Mannheim, Germany). The M2 phenotype macrophages positive with CD163 staining were calculated per field. The CCL2 protein expression was quantified based on the evaluation of staining using semi-quantitative Histoscore (H-score), which was calculated by an assessment of both the intensity of staining (graded as 0, non-staining; 1, weak; 2, median; or 3, strong) and the percentage of positive cells. The range of possible scores was from 0 to 300. The expression level of each component was categorized as low or high according to the H-score's median value. Two independent pathologists blinded to the clinical outcomes performed this analysis.

For immunofluorescence analysis, aHSCs were stained using rabbit anti-human alpha-smooth muscle actin (α-SMA, Abcam, Cambridge, MA, USA) and mouse anti-human CCL2 protein (Sigma Aldrich, St. Louis, MO, USA), followed by Alexa Fluor 555–conjugated donkey anti-mouse IgG and Alexa Fluor 488-conjugated donkey anti-rabbit IgG (Invitrogen, Grand Island, NY, USA). Macrophages were stained using polyclonal mouse anti-human CD163 (DakoCytomation, Glostrup, Denmark). Positive cells were detected using immunofluorescence confocal microscopy (Leica, Germany).

### *In vitro* Co-culture System

The aHSCs were isolated as previously described (32). To minimize the clonal selection and stress during extended culture, the cells were passaged 3–8 times and then used for subsequent experiments. The LX2 cells (hepatic stellate cell line) were obtained from the ATCC cell bank. THP-1 cells (kindly provided by Dr. Songguo Zheng), a human leukemia monocytic cell line, can be differentiated into macrophages under stimulation of 100 ng/ml 12-Otetradecanoylphorbol-l3-acetate (PMA) for 48 h (33, 34). The THP1-derived M0MΦ were cultured in RPMI 1640 containing 10% FBS in 6-well flat-bottomed plates (1 × 10^6^ cells/well) for at least 1 h before co-culture with aHSCs (including primary aHSCs or TGF-β activated LX2 at a ratio of 5:1 or 1:1).

Some reports indicated that TGF-activated LX2 tends to be in a more active status when compared with non-activated LX2. Therefore, we used LX2 and TGF-β activated LX2 as various activation status of aHSCs.

For the supernatant treatment group, we used 50% conditioned supernatant and 50% fresh complete medium to treat the macrophages for 72 h before analysis. When indicated, recombinant human CCL2 protein (rh CCL2, 2ng/ml, R&D Systems, Abingdon, UK) and INCB 3284 (100ng/ml, Tocris Bioscience, UK) were accordingly added, after which the macrophages were harvested, counted, and analyzed.

### Supernatants Preparation and Testing

A total of 5 × 10^5^ aHSCs or 1 × 10^6^ LX2 were seeded per well into 6-well plates containing 2 ml of DMEM with 10% Fetal Bovine Serum (FBS) containing 100 ng/ml benzylpenicillin and 100 ng/ml streptomycin (all purchased from Sigma Aldrich, St. Louis, MO, USA). Once cells reached 90% confluency, the supernatants were harvested, centrifuged, and stored in aliquots at −80°C. The activated LX2 was prepared by stimulation with TGF-β (2 ng/ml, R&D Systems, Abingdon, UK) for no <24 h and then replaced with fresh medium without TGF-β for another 24 h before harvesting the supernatant. The levels of CCL2 in the conditioned supernatants were evaluated using CCL2 enzyme-linked immunosorbent assays (ELISA, R&D Systems, Abingdon, UK) according to the instructions. Next, the macrophages were treated with 50% conditioned supernatant and 50% fresh complete medium for 72 h before analysis.

### Flow Cytometry and Real-Time Quantitative Polymerase Chain Reaction

Monocytes/macrophages (1 × 10^6^) were stained with fluorochrome-conjugated monoclonal antibodies against CD14, CD163, and CD206 (all purchased from BD Biosciences) according to the manufacturer's instructions and then analyzed by flow cytometry. Data were acquired and analyzed on the Flow Cytometer (BD Biosciences). Total RNA was purified from cultured macrophages using TriPure Isolation Reagent Kit (Roche Applied Science, Germany). The cDNA was synthesized using the Transcriptor First-strand cDNA Synthesis Kit (Roche Diagnostics, Mannheim, Germany). Quantitative RT-PCR was performed using SYBR Green PCR kit (Roche Diagnostics, Mannheim, Germany) on the LightCycler 480 apparatus (Roche). The relative concentrations of the target genes were calculated and normalized against that of the housekeeping gene β-actin. Primer sequences are available upon request. The following mRNAs were amplified: CCR2, CD163, ARG1, IL-10.

### Chemotaxis Experiments

Briefly, the upper chamber was seeded with macrophages and separated from a lower chamber. In the lower chamber, aHSCs were seeded with or without CCR2 antagonist INCB 3284. At the specific time point, macrophages that migrated to the underside of the insert were fixed and stained for quantitation by light microscopy. Macrophages were treated with aHSCs, adding INCB plus aHSC, and Rh CCL2; blank medium was used for the control group.

### Statistical Analysis

Statistical analyses were performed using SPSS 22.0. Data conforming to the normal distribution were assessed by independent sample *t*-test and paired *t*-test and expressed as mean ± standard deviation. Non-normally distributed data were compared using the Mann-Whitney *U* test and two-tailed test. *P*-value < 0.05 was considered as statistically significant.

## Results

### Infiltration of M2 Macrophages (CD163^+^) Increased With the Progression of Liver Fibrosis

**Figure 1:**
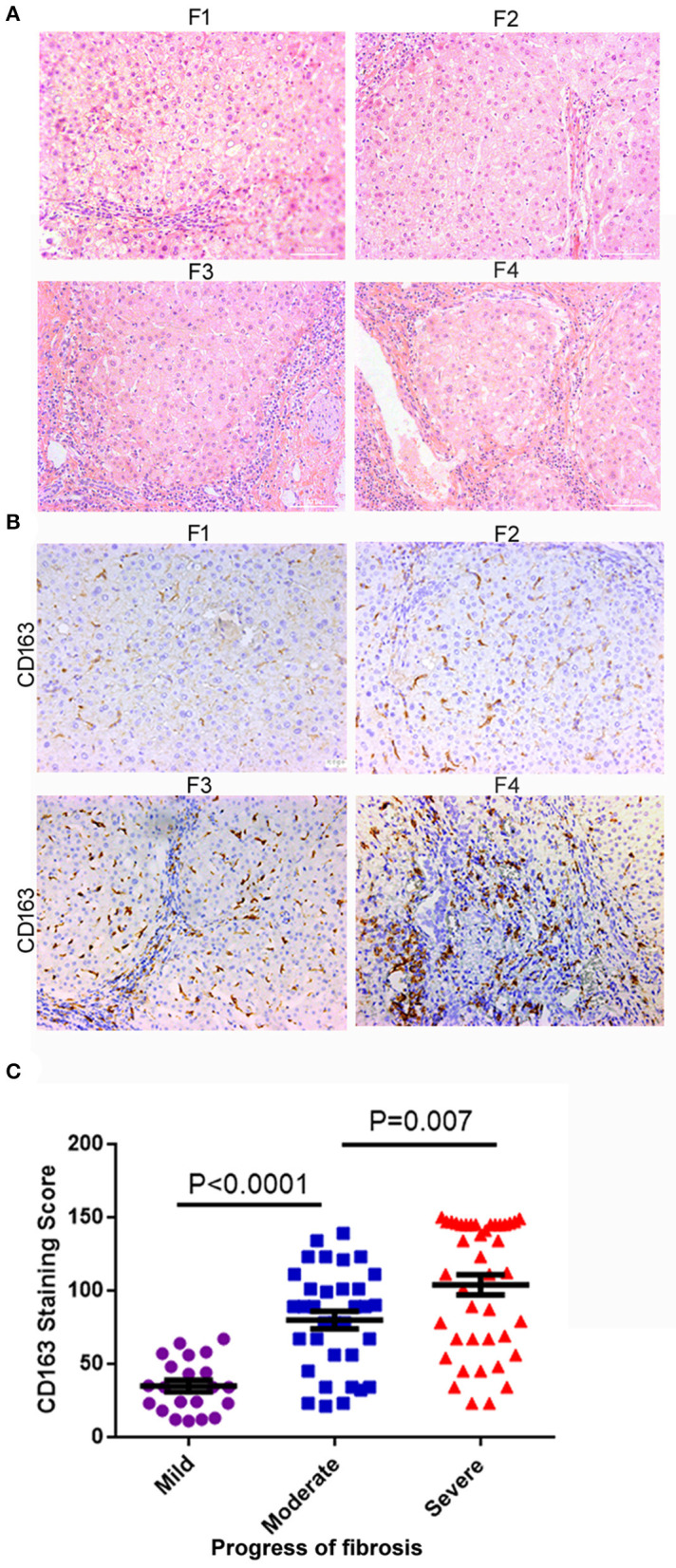
M2 macrophages (CD163+) infiltration increased with liver fibrosis progression. **(A)** The degree of liver fibrosis in different patients was assessed by hematoxylin-eosin staining according to the Metavir score system. F1, fibrosis around the portal vein; F2, fibrous interval around the portal vein; F3, a large number of fibrous intervals are formed between the portal vein and the central vein; F4, cirrhosis. HE staining, the grade of liver fibrosis was based on Metavir score, ×200-fold. **(B)** The expression of CD163 in liver tissues significantly increased as the fibrosis aggravated. **(C)** According to the degree of F0-F4 fibrosis based on the Metavir scoring system, we defined F0-F1, F2-F3, and F4 as mild, moderate, and severe liver fibrosis, respectively. The number of patients in mild degree was 21, in moderate degree was 34, in severe degree was 41. A significant difference was observed between mild and moderate fibrosis (*P* < 0.001), and between moderate and severe fibrosis (*P* = 0.007).

The characteristics of the patients involved in our study are shown in Table 1. To examine the relationship between hepatic M2 macrophages and liver fibrosis stages, we first compared the expression of CD163 (mainly expressed on M2 macrophages) in different stages of hepatic fibrosis among patients. The degree of liver fibrosis in different patients was assessed by hematoxylin-eosin staining according to the Metavir score system (F0, no fibrosis; F1, fibrosis around the portal vein; F2, fibrous interval around the portal vein; F3, a large number of fibrous intervals are formed between the portal vein and the central vein; F4, cirrhosis) (Figure 1A). F0 and F1 were defined as mild fibrosis, F2 and F3 as moderate fibrosis, and F4 as severe fibrosis. As shown in Figures 1B,C, the expression of CD163 in liver tissues significantly increased with fibrosis. The number of patients in mild degree was 21, in moderate degree was 34, in severe degree was 41. The CD163 IHC score in the mild degree was 34.95 ± 18.12, in moderate degree was 77.57 ± 32.48, and in severe degree was 99.62 ± 40.84; a significant difference was observed between mild and moderate fibrosis (*P* < 0.001), and between moderate and severe fibrosis (*P* = 0.007). These results indicated the correlation between M2 macrophages and fibrosis. We concluded that the fibrosis environment might affect the phenotype of macrophages.

**Table 1:** Basic Clinical Characteristics of the Study Population (*n* = 96).

**Variables**	**Patients, *N* (%)**
**Gender**	
Female	15(15.63)
Male	81(84.37)
**Age (years)**	
≤40	15(15.63)
41–50	22(22.92)
51–60	36(37.5)
61–70	19(19.79)
>70	4(4.16)
**ALT (U/L)**	
≤40	58(60.42)
>40	37(30.58)
**AST(U/L)**	
≤40	55(57.29)
>40	41(42.71)
**ALB/GLB**	
>2.5	2(2.08)
1.0–2.5	91(94.79)
<1.0	3(3.13)
**Total bilirubin(μmol/L)**	
≤17	67(69.79)
>17	29(30.21)
**HBeAg**	
(+)	17(17.71)
(–)	75(78.13)
None	4(4.17)
**HBV DNA**	
<10^4^	37(38.54)
10^4^–10^6^	29(30.21)
≥10^6^	18(18.75)
None	12(12.5)
**Platelets (10**^**9**^**/L)**	
Median (IQR)	181.5(148.75–181.5)
**White blood cells (10**^**9**^**/L)**	
Median (IQR)	6.01(4.74–8.14)
NEUT%	59.00(50.50–68.25)
MO%	8.00(7.00–10.00)
LYM%	29.00(19.75–36.25)
**Fibrosis**	
F0-1(Mild)	21(21.88)
F2-3 (Moderate)	34(35.41)
F4(Severe/cirrhosis)	41(42.71)

*ALT, alanine aminotransferase; AST, aspartate aminotransferase; ALB, albumin; GLB, globulin*.

### M0MΦ Developed into M2 Phenotype When Co-cultured With aHSCs or Treated With Supernatant From aHSCs

**Figure 2:**
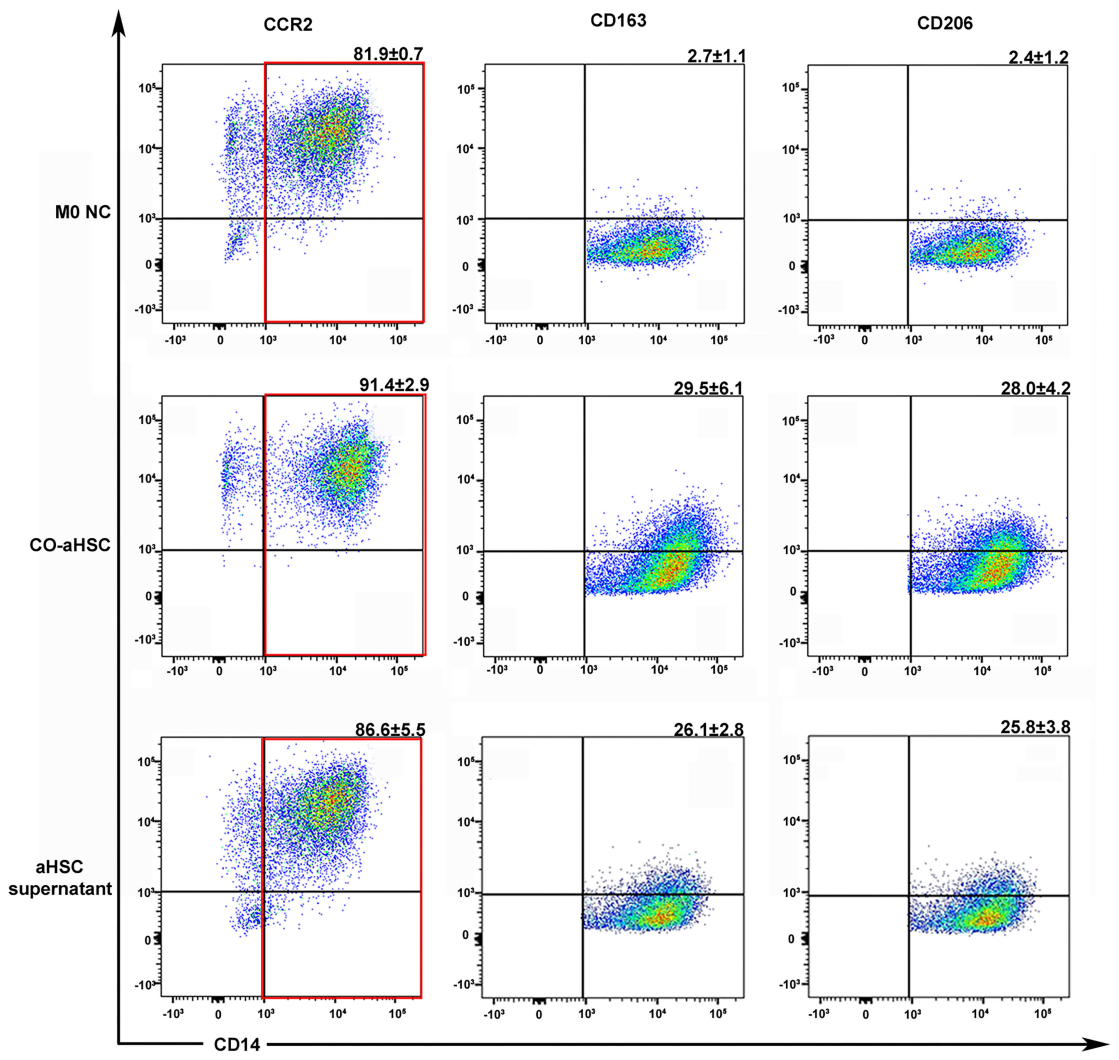
Either the co-culture condition with aHSCs or their supernatant could independently induce the M2 macrophage differentiation. After 5 days culture, compared to the control group (M0 NC), the co-aHSC group highly expressed M2 phenotype specific proteins: CD163 (29.5 ± 6.1% vs. 2.7 ± 1.1%, *P* < 0.001) and CD206 (28.0 ± 4.2% vs. 2.4 ± 1.2%, *P* < 0.001). The aHSC supernatant group independently up-regulated the expression of CD163 and CD206 on macrophages as compared to the M0 NC group (26.1 ± 2.8% vs. 2.7 ± 1.1%, *P* < 0.001 and 25.8 ± 3.8% vs. 2.4 ± 1.2%, *P* < 0.001). These experiments were repeated at least three times.

Hepatic stellate cells are always activated during liver fibrosis and can secrete many cytokines and chemokines (23). Thus, whether aHSCs are responsible for the monocyte infiltration and M2 phenotype formation in livers is yet to be elucidated. We successfully extracted five cases of primary human aHSCs with sustainable and stable growth. To investigate the regulation of aHSCs on macrophages, the primary aHSCs were co-cultured with THP-1-derived M0MΦ (1:5 cell ratio). After 5 days of co-culture, as shown in Figure 2, compared with the M0 control group, the aHSCs group significantly up-regulated the expression of CD163 and CD206 on macrophages: (29.5 ± 6.1% vs. 2.7 ± 1.1%, *P* < 0.001 and 28.0 ± 4.2% vs. 2.4 ± 1.2%, *P* < 0.001). This suggested that aHSCs can regulate macrophages through certain pathways, promoting the M2 phenotype differentiation.

To further explore the mechanism of the immunomodulatory effects of aHSCs on macrophages, we detected the phenotypic changes in M0MΦ after treating with aHSCs' supernatant. As shown in Figure 2, the supernatant treated group independently up-regulated the expression of CD163 and CD206 on macrophages compared with the control group (26.1 ± 2.8% vs. 2.7 ± 1.1%, *P* < 0.001 and 25.8 ± 3.8% vs. 2.4 ± 1.2%, *P* < 0.001), indicating that the aHSCs might secrete specific cytokines responsible for the macrophages' phenotype transformation. Of note, macrophages in the supernatant group showed relatively lower expression of CD163 and CD206 compared with the co-culture group, indicating that cell to cell contact might also induce other ways to promote M2 phenotype transformation besides soluble molecules.

### AHSCs Secrete High Levels of CCL2

In a previous study, we found that aHSCs secrete various cytokines and chemokines, including CCL2 (23). CCL2 can recruit immune cells, including monocytes. However, whether it participates in up-regulating the expression of CD163 and CD206 on macrophages is yet to be elucidated.

**Figure 3:**
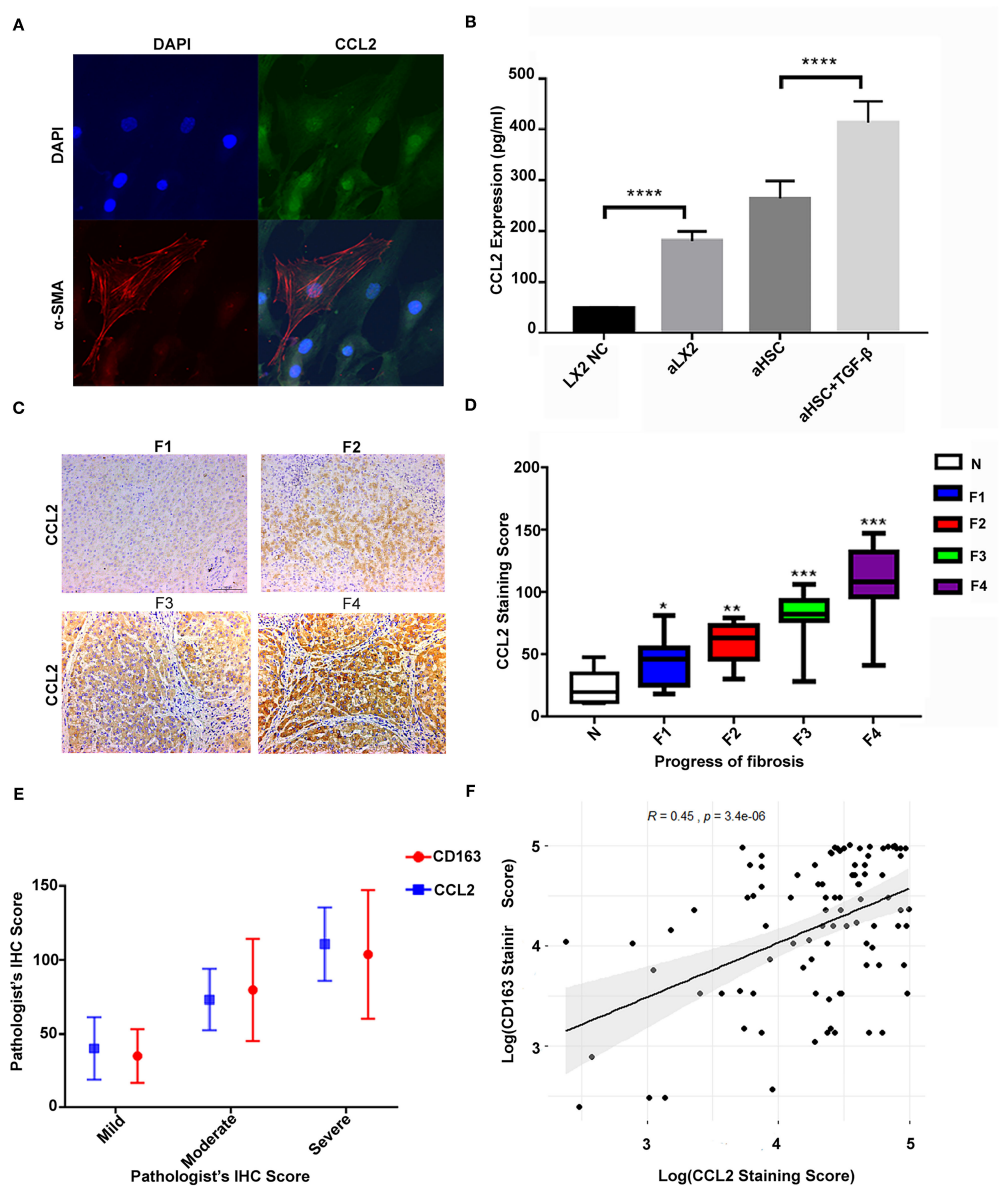
The high level of CCL2 in aHSCs was associated with CD163+ macrophage infiltration and increased with liver fibrosis progression. **(A)** The primary aHSCs are typically fusiform and express the activation marker α-SMA, together with a high expression of CCL2 protein. **(B)** The aHSCs secrete high levels of CCL2 as compared to LX2, which is further enhanced by TGF-β stimulation (LX2 vs. TGF-β-stimulated LX2, *P* < 0.001; aHSC vs. TGF-β-stimulated aHSCs, *P* < 0.001). **(C)** Representative figures for immunohistochemistry staining of liver tissues with antibody of CCL2. As the degree of liver fibrosis worsened (F1 to F4), the expression of CCL2 increased gradually. **(D)** Comparison of CCL2 staining score among different fibrotic status. The number of patients for *N* was 7, F1 was 14, F2 was 12, F3 was 14, and F4 was 41. **(E)** The correlations between CD163 and CCL2 were explored under different fibrotic degree. **(F)** A relatively strong correlation was established between M2 macrophage (CD163+) IHC staining score and CCL2 score by scatter plot (*R* = 0.45, *P* < 0.05).

First, we confirmed the production of CCL2 in aHSCs as well as in those stimulated by TGF-β. To compare the results, we used the LX2 cell line as the control. Figure 3A showed that the primary aHSCs are typically fusiform and express the activation marker α-SMA together with a high expression of the CCL2 protein. The expression of CCL2 in the supernatant of the primary aHSC, TGF-β (2 ng/ml)-stimulated aHSCs, LX2, and TGF-β (2 ng/ml)-stimulated LX2 was evaluated by ELISA. As shown in Figure 3B, aHSCs can secrete high levels of CCL2 compared to LX2. Interestingly, this ability can be further enhanced by TGF-β stimulation (LX2 vs. TGF-β stimulated LX2, P < 0.001; aHSC vs. TGF-β stimulated aHSCs, *P* < 0.001).

Furthermore, in consistent to what we found in the *in vitro* study, the expression of CCL2 increased with liver fibrosis progress. As shown in Figures 3C,D, as the degree of liver fibrosis worsened, the expression of CCL2 gradually increased as compared to the control group (N group) (F1 vs. N, *P* = 0.013, F2 vs. N, *P* < 0.01, F3 vs. N, *P* < 0.001, F4 vs. N, *P* < 0.001). The number of patients for N was 7, for F1 was 14, F2 was 12, F3 was 14, and F4 was 41. The CCL2 staining score for N was 23.26 ± 13.85; for F1 was 48.56 ± 19.18 (F1 vs. N, *P* = 0.013); for F2 was 58.25 ± 16.24 (F2 vs. N, *P* < 0.01); F3 was 81.33 ± 18.48 (F3 vs. N, *P* < 0.001); F4 was 110.93 ± 24.75, (F4 vs. N, *P* < 0.001). Further statistical analyses were also performed for possible correlation among CD163, CCL2 as well as their correlation with other clinical variables (Table 2). These data indicated CCL2 was significantly related to lymphocytes' levels. Noteworthy, both CCL2 and CD163 staining scores have a statistically significant correlation with the ALB/GLB ratio.

**Table 2:** Correlation analysis between clinical characteristics and CD163 and CCL2.

**Demographic or Characteristics**	**CD163**		**CCL2**	
	***R***	***P*-value**	***R***	***P*-value**
Age (years)	0.074	0.473	0.158	0.123
ALT (U/L)	0.113	0.276	0.085	0.416
AST (U/L)	0.103	0.325	0.101	0.332
Total bilirubin (μmol/L)	0.130	0.215	0.037	0.721
ALB/GLB	−0.299	0.003*	−0.445	<0.001**
White blood cells (10^9^/L)	−0.019	0.859	0.027	0.795
NEUT%	−0.024	0.816	−0.029	0.784
MO%	0.060	0.565	0.107	0.303
LYM%	−0.001	0.989	0.397	<0.001**
Platelets (10^9^/L)	−0.043	0.683	−0.074	0.477

*P-value ≤ 0.05; ***P-value ≤ 0.001.

*r: Pearson correlation coefficient*.

*ALT, alanine aminotransferase; AST, aspartate aminotransferase; ALB, albumin; GLB, globulin*.

Additionally, a significant increase in both markers (CCL2 and CD163) was detected with the advancement in fibrosis. The correlations between CD163 and CCL2 were explored under different fibrotic degree and by scatter plot (Figures 3E,F), which suggested a correlation between the IHC score of M2 macrophage (CD163+) and CCL2 (*R* = 0.45, *P* < 0.05). These results strengthened our hypothesis that CCL2 may regulate M2 phenotype transformation.

### aHSCs Induce Macrophage Infiltration and M2 Differentiation *via* CCL2

**Figure 4:**
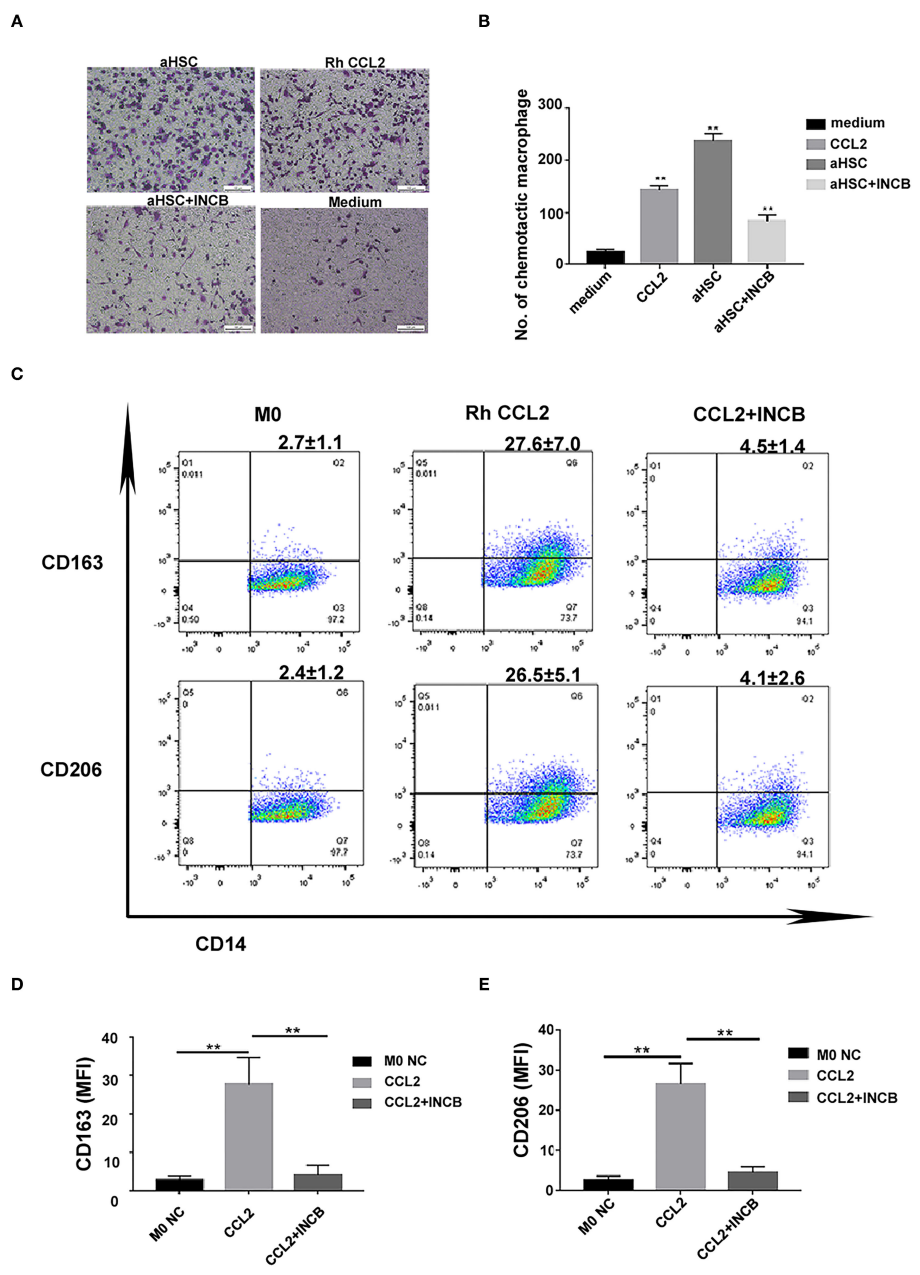
CCL2 was responsible for macrophages infiltration and differentiation into M2 phenotype during liver fibrosis. **(A)** Representative images of macrophage infiltration under different chemotaxis treatments including aHSC, aHSC+INCB, Rh CCL2 and medium. **(B)** Statistical analysis of the number of macrophages infiltration. The aHSC group vs. medium group, *P* < 0.01, aHSC+INCB group vs. aHSC group, *P* < 0.01. Rh CCL2 vs. medium group, *P* < 0.01. **(C)** The representative flow cytometry data of macrophage phenotypic change when exposed to CCL2 treatment with or without INCB. **(D)** CCL2 significantly up-regulated CD163 expression on macrophages; this effect could be blocked by INCB (100 ng/ml). (CCL2 group vs. M0 NC group, *P* = 0.008; CCL2 group vs. CCL2 + INCB group, *P* < 0.01). **(E)** CCL2 significantly up-regulated CD206 expression on macrophages, and this effect could be blocked by INCB (100 ng/ml). (CCL2 group vs. M0 NC group, *P* = 0.003; CCL2 group vs. CCL2 + INCB group, *P* < 0.01). These experiments were repeated at least three times.

To verify that CCL2 was responsible for macrophage infiltration and differentiation into M2 phenotype during liver fibrosis, we used rh CCL2 and its receptor antagonist INCB to confirm this pathway further. As shown in Figures 4A,B, macrophage infiltration increased when aHSCs were cultured in the lower chamber as compared to the control (only medium) (aHSC group vs. medium group, 237.00 ± 13.78 vs. 23.75 ± 5.32, *P* < 0.01). In contrary, the number decreased when INCB was used to block the CCL2/CCR2 pathway in the same culture system (aHSC + INCB group vs. aHSC group 83.25 ± 12.78 vs. 237.00 ± 13.78, *P* < 0.01). Rh CCL2 could also mimic aHSCs on the ability of macrophage infiltration (Rh CCL2 group vs. medium group, 143.25 ± 7.80 vs. 23.75 ± 5.32, *P* < 0.01), indicating that aHSCs promote macrophage infiltration mainly through CCL2. The experiment was repeated four times.

Furthermore, CD163 and CD206 expression was significantly up-regulated on macrophages as a result of rh CCL2 stimulation as compared to the control group. The flow cytometry result of CCL2 is shown in Figures 4C,E (CCL2 vs. M0, CD163: 27.6 ± 7.0% vs. 2.7 ± 1.1%, *P* = 0.008; CD206: 26.5 ± 5.1% vs. 2.4 ± 1.2 %, *P* = 0.003). The addition of INCB (100 ng/ml) inhibited the expression of CD163 and CD206 on M0MΦ (CD163: 4.5 ± 1.4%, CD206: 4.1 ± 2.6%, vs. M0 NC group, *P* > 0.05).

**Figure 5:**
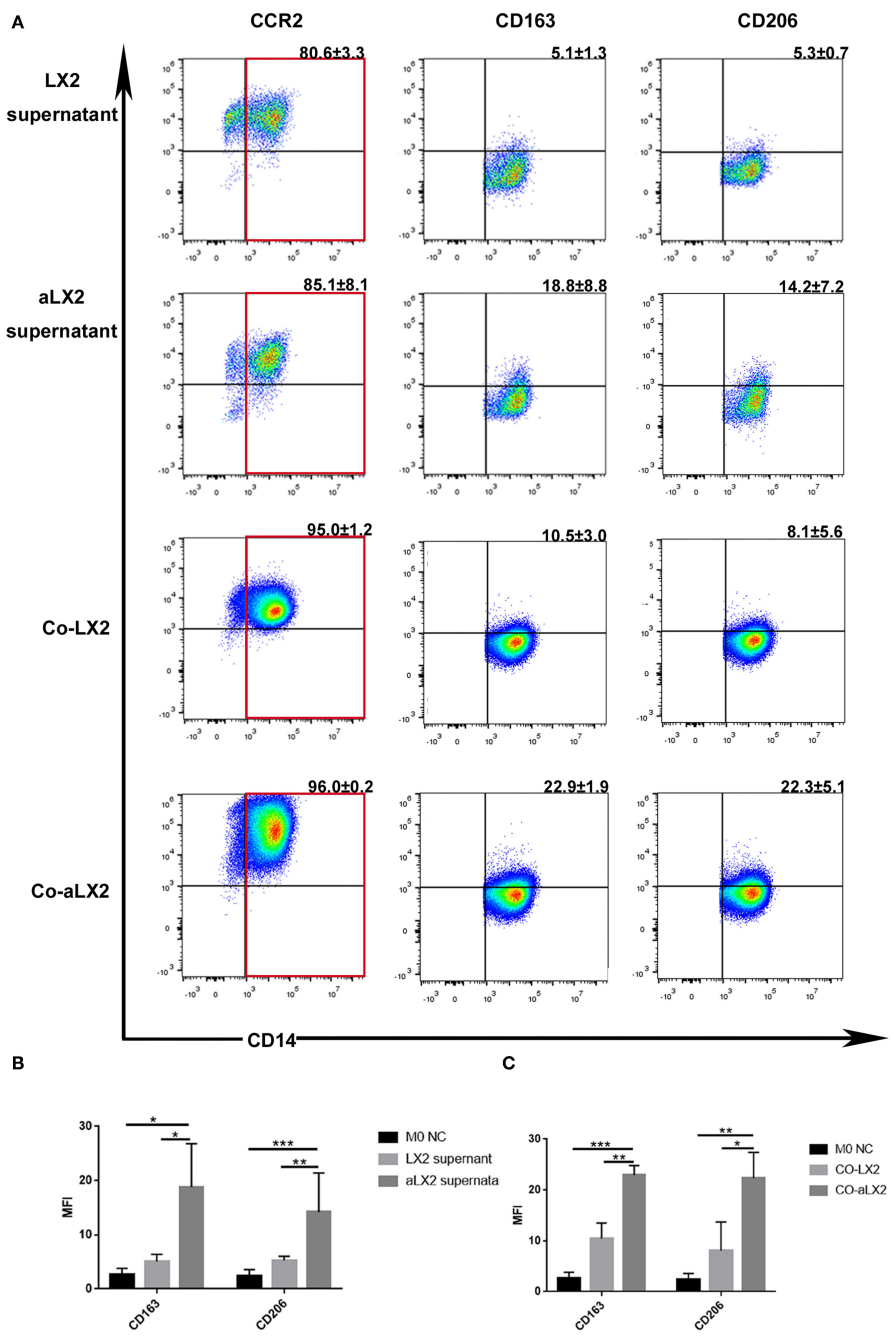
TGF-β-stimulated LX2 up-regulated the expression of CD163 and CD206 on macrophages under co-culture or supernatant treatment condition. **(A)** The representative flow cytometry figure of macrophage phenotypic change when exposed to co-culture with LX2 or TGF-β stimulated LX2 (aLX2) or their supernatant treatment. **(B)** The aLX2 supernatant group independently up-regulated the expression of CD163 and CD206 on macrophages. (CD163: aLX2 group vs. LX2 group, *P* < 0.05), (CD163: aLX2 group vs. M0 NC group, *P* < 0.05); (CD206: aLX2 group vs. LX2 group, *P* < 0.05), (CD206: aLX2 group vs. M0 NC group, *P* < 0.05). **(C)** The co-culture with aLX2 independently up-regulated the expression of CD163 and CD206 on macrophages. CD163 (aLX2 group vs. LX2 group, *P* < 0.05; aLX2 group vs. M0 NC group, *P* < 0.05) and CD206 (aLX2 group vs. LX2 group, *P* < 0.05; aLX2 group vs. M0 NC group, *P* < 0.05). These experiments were repeated at least three times.

To avoid the clonal selection and individual differences of the primary aHSCs, we used LX2 cell lines to repeat the experiment. Treatment of LX2 cells with TGF-β induces proliferation and expression of ECM components. Previous studies have shown that the stimulation with TGF-β (2 ng/ml) for 24–48 h increased mRNA and protein expression of alpha-SMA, collagen type 1, PDGF, TIMP1, and TIMP2 compared to untreated LX2 cells (34, 35). Other studies have reported similar trends (36–39), thus suggesting that TGF-β activates LX2 cells to a greater extent than simple culturing of cells on a stiff surface. Compared to the LX2 group, TGF-β-stimulated LX2 (aLX2 group) up-regulated the expression of CD163 and CD206 on macrophages in both the co-culture system and when using supernatant only (Figure 5). Since activated LX2 also produced large amounts of CCL2 (Figure 3B) and CCL2-specific receptor antagonist INCB blocked the macrophages' modulation function (Figures 4C–E), we concluded that aHSCs induce macrophage M2 phenotype differentiation through the CCL2/CCR2 pathway.

### aHSCs Upregulated the Expression of CCR2 to Form CCL2/CCR2 Positive Feedback Pathway

Interestingly, while we used qPCR to test the mRNA levels of M2MΦ-specific markers after treating THP-1-derived M0MΦ under different conditions (supernatants stimulation from aHSC, aLX2, LX2), we found that supernatants from aHSCs up-regulated the expression of macrophage CCR2, ARG-1, and IL-10 in addition to CD163 (Supplementary Figure 1). Furthermore, we confirmed the upregulation of CD163 on CCL2-stimulated macrophages by immunofluorescence (Supplementary Figure 1B). In conclusion, these results indicated that besides the secretion of high levels of CCL2, aHSCs could also up-regulate the expression of CCR2 on macrophages to activate the CCL2/CCR2 pathway. However, the mechanism needs further exploration.

## Discussion

The M2 macrophage infiltration increased significantly with the progression of liver fibrosis, accompanied by the up-regulated expression of CCL2 in the fibrotic liver tissues. *In vitro* data showed that primary aHSCs isolated from fibrotic liver tissues and aLX2 could produce CCL2, promoting macrophages' conversion to the M2 phenotype, which displays its immunosuppressive function by secreting the inhibitory cytokines such as IL-10 and ARG1. This might contribute to an immunosuppressive state in the liver. It may also explain an increased risk of hepatocellular carcinoma and bacterial infection in cirrhotic patients (17, 40).

CD163 is a multifunctional receptor involved in receptor-mediated endocytosis and signal pathways upon interaction with diverse ligands. CD163-positive macrophages are usually found during the healing phase of acute inflammation and chronic inflammation in wound-healing tissues, whereas freshly infiltrated macrophages are CD163-negative (41). CD163 acts as an innate immune sensor for bacteria and inducer of local immunity, rather than as a phagocytic receptor, and has been proposed as an anti-inflammatory marker for macrophages (42). CD163 is shed from the macrophage surface into the circulation upon activation of cell surface Toll-like receptors (TLRs) and is found in the blood as soluble CD163 (sCD163). Previous studies reported increased sCD163 levels in patients with chronic viral hepatitis; these increased levels have been associated with the disease's severity and may predict fibrosis (43–45). It is also determined that the sCD163 serum level is a new independent non-invasive risk factor for death and variceal bleeding in cirrhotic patients (46). However, the link between hepatic CD163^+^ macrophages accumulation and liver fibrosis progression remain elusive, and experimental evidence for the reasons why CD163 upregulation during liver fibrosis is still lacking. In this study, we found that the expression of CD163 in liver tissues significantly increased as the fibrosis aggravated. This may explain the increase of sCD163 levels in patients' serum as sCD163 is discarded from activated macrophages, which are mainly located in the liver.

Interestingly, we found that CCL2 improve THP1-derived M0MΦ differentiation into CD163^+^ macrophages independently. We speculated that, besides the recruitment of immune cells, CCL2 might also play immune modulation roles. Indeed, we discovered that the supernatant from aHSCs up-regulated the expression of CD163 on macrophages more obviously than CCL2 alone, which indicated that other factors in aHSCs supernatant might also induce the expression of CD163. It has been reported that the expression of CD163 could be induced by glucocorticoids, IL-10, IL-6, and M-CSF (41). In our previous study, we found that aHSCs can secrete high levels of IL-6 and M-CSF (23), which may explain why aHSCs showed stronger up-regulation function than Rh CCL2 alone. It also indicated complicated immune modulation pathways during liver fibrosis where aHSCs dominate.

Recent studies showed that the CCL2/CCR2 axis has a vital role in the fibrotic formation in some diseases, including pulmonary fibrosis, renal fibrosis, and non-alcoholic liver fibrosis. In addition, it also regulates chemotactic macrophage infiltration (27, 47, 48). Wang et al. speculated that the CCL2/CCR2 axis is closely related to the aggregation of myeloid-derived inhibitory cells (MDSCs) and T cell function inhibition in pulmonary fibrosis and lung cancer (49). The proportions of CD11C^+^CD206^+^ and CCR2^+^ macrophages in adipose tissues were highly elevated in patients with NASH compared to healthy controls and patients with fatty liver, and CCR2^+^ macrophages were also correlated with NASH severity (44). It seems that CCR2^+^ macrophages have essential roles in inflammation, cancer, and fibrosis. Still, only a few studies examined why and how CCR2 are up-regulated on macrophages. The current study revealed the correlation between the high expression of CCL2, macrophage infiltration, and the hepatic fibrosis progress; the level of CCL2 strikingly increased with continuous activation of HSCs, which is essential in cirrhosis. Some factors from aHSCs may help to stimulate CCR2 expression. This may provide some clues and explanations to the CCR2^+^ macrophages recruitment that have not been addressed by previous studies.

In the cell culture system, our results showed that aHSCs induce macrophages aggregation and M2 phenotype differentiation through CCL2/CCR2. On one hand, M2 macrophages might accelerate the immune surveillance disorder under cirrhotic condition by secreting immunosuppressive cytokines (5), showing a relatively weak antigen presentation, promoting vascular regeneration and tissue reconstruction (13). On the other hand, M2 macrophages secrete IL-10, TGF-β, and other cytokines, which are beneficial to the survival and sustained activation of aHSCs (40). Therefore, we propose that during the progression of liver fibrosis (especially HBV related), there might be an “amplification loop” between aHSCs and macrophages, and CCL2/CCR2 axis has essential roles in this loop. Since a study found some anti-fibrotic effects of CCL2 inhibitor in animal models of liver fibrosis (30), the pathway of CCL2/CCR2 as potential therapeutic targets should be further investigated by future studies.

The present study has some limitations. Firstly, due to the limited experiment conditions, we could not confirm our results in animal models, especially transgenic mice models. We plan to further investigate the detailed mechanisms *in vivo* in our next study. Secondly, although some experiments on CD163 and CCR2+ macrophages gave us indications that this kind of macrophages might contribute to immune suppression status in the liver and might help to develop HCC and bacterium infection (40), we do not have robust data and direct evidence to prove these hypothesis. Thirdly, a detailed mechanism such as a signaling pathway in the CCL2/CCR2 axis mediated the activation in macrophages should be investigated by future studies. Lastly, all our *in vitro* experiments were based on the THP-1 cells, a human leukemia monocytic cell line. We are not sure if this reflects the same situation in that of the primary normal human monocytes. It is warranted to repeat all the experiments with primary human monocytes if possible.

In conclusion, we found increased expression of hepatic CD163 and CCL2 in patients with HBV-related fibrosis; the expression increased dramatically with further progression of liver fibrosis. There might be an “amplification loop” between aHSCs and macrophages through CCL2/CCR2 axis; thus, more *in vitro* and *in vivo* studies in this area are needed.

## Data Availability Statement

The original contributions presented in the study are included in the article/Supplementary Material, further inquiries can be directed to the corresponding author/s.

## Ethics Statement

The studies involving human participants were reviewed and approved by Institutional Review Board of the Third Affiliated Hospital of Sun Yat-sen University. Written informed consent for participation was not required for this study in accordance with the national legislation and the institutional requirements.

## Author Contributions

MZ, YC, SX: conception and design. MZ and YC: financial support. SX and XL: experiment conduction. SX and XL: provision of materials or patients. SX, XZ, YJ, ZL, and JC: collection and assembly of data. SX, XZ, YW, and JG: data analysis and interpretation. XZ: revisional author. All authors: manuscript writing. All authors: final approval of manuscript.

## Conflict of Interest

The authors declare that the research was conducted in the absence of any commercial or financial relationships that could be construed as a potential conflict of interest.
