# Supplementary material for: Activated Hepatic Stellate Cells Induce Infiltration and Formation of CD163+ Macrophages via CCL2/CCR2 Pathway
Source: Front Med (Lausanne). 2021 Feb 5;8:627927. doi: 10.3389/fmed.2021.627927 (PMC7893116; doi:10.3389/fmed.2021.627927)
Supplement: Supplementary file 1 [file Table_1.DOCX]

**Supplementary Table 1 Primers used during the study**

| β-actin: | F:GCACCCAGCACAATGAAGAT  R:ACATCTGCTGGAAGGTGGAC |
| --- | --- |
| CD163: | F: CAATGAAGATGCTGGCGTGA  R: CCTGCAAACCACATCAGCTT |
| IL-10: | F: ACATCAGGGGCTTGCTCTT  R: CTCGAAGCATGTTAGGCAGG |
| ARG1: | F: CTGTGGGAAAAGCAAGCGAG  R: CACTTGTGGTTGTCAGTGGA |
| CCR2: | F: CCACATCTCGTTCTCGGTTTATC |

**Supplementary Figure 1. AHSCs up-regulated macrophage CCR2 expression to form CCL2/CCR2 positive feedback circle pathway. A.** M2MΦ specific markers expression in mRNA levels after treating THP-1 derived M0MΦ at different conditions (supernatants stimulation from aHSC, aLX2, LX2, and complete medium) were tested by qPCR. The results showed that CCL2 up-regulated macrophage CCR2, ARG-1, and IL-10 expression in addition to CD163 at the gene level. **B.** The co-localization of CD163 and CCR2 on CCL2 stimulated macrophages by immunofluorescence.
