# Supplementary figures and images for: Activated Hepatic Stellate Cells Induce Infiltration and Formation of CD163+ Macrophages via CCL2/CCR2 Pathway
Source: Front Med (Lausanne). 2021 Feb 5;8:627927. doi: 10.3389/fmed.2021.627927 (PMC7893116; doi:10.3389/fmed.2021.627927)

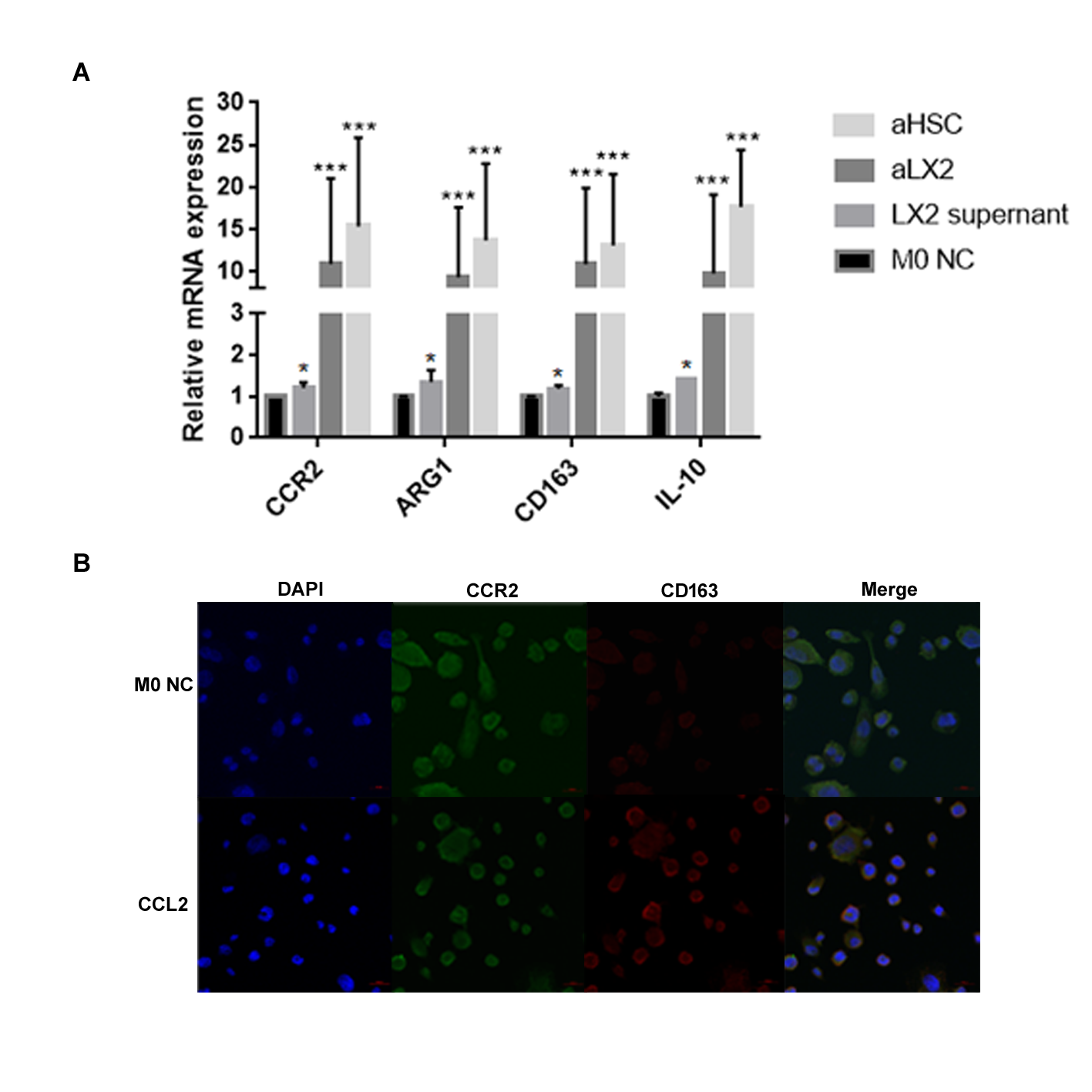

Supplement: Supplementary file 2 [file Image_1.TIF]
